# Comparison and evaluation of metagenomic next-generation sequencing (mNGS) and real-time PCR for the detection of *Mycobacterium tuberculosis*

**DOI:** 10.3389/fcimb.2025.1694179

**Published:** 2025-11-07

**Authors:** Yanhong Wang, Penghao Guo, Yaoming Chen, Hongji Zhu, Xuegao Yu, Jiankai Deng

**Affiliations:** Department of Laboratory Medicine, The First Affiliated Hospital, Sun Yat-Sen University, Guangzhou, China

**Keywords:** metagenomic next-generation sequencing, real-time PCR, tuberculosis diagnosis, pulmonary tuberculosis, extrapulmonary tuberculosis

## Abstract

**Objective:**

This study aimed to evaluate and compare the performance of metagenomic next-generation sequencing (mNGS) and real-time polymerase chain reaction (RT-PCR) for the detection of *Mycobacterium tuberculosis* (MTB) in patients with suspected tuberculosis (TB).

**Methods:**

Samples from patients undergoing routine clinical testing for MTB using both mNGS and RT-PCR were included. The diagnostic agreement between the two methods was assessed. Discordant results were further validated using the Xpert MTB/RIF assay on cryopreserved aliquots.

**Results:**

A total of 556 samples from suspected TB patients were analyzed. The majority were lower respiratory tract specimens, including bronchoalveolar lavage fluid (BALF; 94.06%), sputum (3.24%), and extrapulmonary samples (2.70%). Compared with Xpert MTB/RIF and clinical diagnosis as composite reference standard, both mNGS and RT-PCR showed high sensitivity (92.31% and 90.38%, respectively) and perfect specificity (100%). There was a high level of agreement between mNGS and RT-PCR, with a positive agreement of 82.69%, negative agreement of 98.25%, overall agreement of 98.38%, and a *kappa* value of 0.896 (*P* < 0.001). Concordance was higher in samples with lower RT-PCR cycle threshold (Ct) values: 100% at Ct ≤ 15, 100% at 15<Ct ≤ 20, 76.47% at 20<Ct ≤ 25. Among concordant positive samples (n=43), mNGS SMRNs showed a strong negative correlation with RT-PCR Ct values (*r* = -0.668, *P* < 0.001). Analysis of nine discordant cases identified five mNGS-positive/RT-PCR-negative samples with low SMRNs (median: 7 vs. 1788 in concordant positives), four of which were confirmed by Xpert MTB/RIF to have extremely low bacterial loads. The remaining four mNGS-negative/RT-PCR-positive samples exhibited higher Ct values (median: 22.97 vs. 17.06 in concordant positives), and three of these were also verified by Xpert MTB/RIF to contain extremely low bacterial concentrations.

**Conclusion:**

Both RT-PCR and mNGS demonstrate high overall agreement for MTB detection, with concordance strongly influenced by microbial burden. These findings support the complementary use of these methods in the diagnosis of TB.

## Introduction

Tuberculosis (TB), caused by *Mycobacterium tuberculosis* (MTB), remains a life-threatening infectious disease with a profound global health impact. In 2023, an estimated 10.8 million individuals developed TB, and 1.25 million died from the disease, making it the top infectious disease-related cause of mortality worldwide (Global tuberculosis report, 2024). China accounts for 6.8% of the global TB incidence, ranking third among the 30 high-TB-burden countries. Effective TB control relies heavily on early and accurate diagnosis to facilitate timely treatment and interrupt disease transmission.

Current diagnostic standards for TB depend largely on etiological confirmation. Conventional techniques such as acid-fast bacilli (AFB) microscopy and culture remain widely used. Although AFB microscopy is rapid and cost-effective, its limited sensitivity results in a high rate of false-negative diagnoses (Liu et al., 2021; Liu et al., 2023). Moreover, it cannot differentiate between MTB and nontuberculous mycobacteria (NTM) (Dong et al., 2022). Culture, while considered the gold standard for its superior sensitivity and specificity and its ability to support drug susceptibility testing, is significantly constrained by prolonged turnaround times—typically ranging from 2 to 8 weeks—which delays clinical decision-making (Liu et al., 2021; Sam et al., 2021).

In response to these limitations, the World Health Organization (WHO) updated its guidelines in 2013 to include molecular methods as part of the etiological confirmation of TB. Among these, loop-mediated isothermal amplification (LAMP), a diagnostic technique widely applied in infectious disease diagnostics (Liu, 2025), was recommended by the WHO in 2016 as an alternative to sputum smear microscopy for diagnosing patients with suspected TB. This approach is particularly valuable in resource-limited settings, where it can also serve as a follow-up test for adults presenting with signs and symptoms of pulmonary TB—especially for AFB-negative specimens that require further confirmation (Inbaraj et al., 2025). In parallel, real-time polymerase chain reaction (RT-PCR) has become the most widely adopted molecular technique. It employs MTB-specific primers and fluorescent probes (e.g., TaqMan) to enable real-time amplification monitoring, offering high specificity without cross-reacting with NTM. RT-PCR delivers results within hours and exhibits better sensitivity and specificity than conventional smear microscopy and culture (Babafemi et al., 2017). In recent years, several automated, low-complexity nucleic acid amplification tests have further streamlined molecular TB diagnosis.

Metagenomic next-generation sequencing (mNGS) is an emerging culture-independent diagnostic tool that enables comprehensive pathogen detection by sequencing all nucleic acids in a clinical sample and analyzing them through bioinformatic pipelines (Zhao et al., 2024). This method allows for the identification of MTB regardless of bacterial viability and does not require species-specific primers. Additionally, mNGS can concurrently detect NTM and other pathogens including bacteria, fungi, viruses and parasites (Wang and Xing, 2023). Although mNGS has shown revolutionary potential in infectious disease diagnostics and is increasingly used in clinical settings (He et al., 2023), its practical utility and optimal scope of application require further validation.

While numerous studies have individually evaluated the diagnostic performance of mNGS and RT-PCR for TB (Wei et al., 2019; Shi et al., 2020; Babafemi et al., 2021; Gao et al., 2024), there remains a lack of large-scale clinical comparisons directly assessing their agreement in detecting MTB. Evaluating the concordance between these two methods is clinically essential, as it influences the interpretation of discordant results and guides diagnostic strategy selection. Through a retrospective analysis, this study aims to compare the diagnostic accuracy of mNGS and RT-PCR for TB and to evaluate their agreement, thereby providing evidence-based insights to support clinical practice.

## Methods

### Study design and population

This study employed a retrospective analytical methodology. We collected clinical data from a total of 556 suspected TB patients admitted to the First Affiliated Hospital of Sun Yat-sen University between July 2024 and March 2025. All enrolled patients underwent both mNGS and RT-PCR assays for MTB detection on samples obtained concurrently from the identical anatomical site. We simultaneously extracted patients’ baseline data (including demographic information and underlying diseases/comorbidities) and clinical diagnosis from electronic medical records. To resolve discrepancies between the two methods, aliquots of mNGS samples stored at -80 °C were used for subsequent Xpert MTB/RIF assays on discordant specimens.

### Metagenomic next-generation sequencing

The specific procedures for mNGS were performed as described in prior studies (Chen et al., 2023). The DNA was extracted using the IDSeq™ Micro DNA Kit (Vision Medicals Technology Co., Ltd. Guangzhou, China). After purification and fragment selection, constructed the DNA library using the transposase method; performed 75 bp single-end sequencing on the Illumina NextSeq 550 platform, ensuring each sample contained over 10 million reads with a quality score of 30 (Q30) ≥85%. Included negative controls in each batch to monitor contamination. Used fastp software to filter out low-quality and short sequences (length <35 bp), and removed human genome sequences (GRCh38) through BWA alignment; aligned the remaining data against pathogen databases. Pathogens were determined by applying a strict mapped read number (standardized microbial read numbers, SMRNs), defined as the number of sequencing reads precisely aligned to the reference genome or designated genomic regions, with SMRNs ≥1 set for MTB.

### Real-time fluorescence polymerase chain reaction

Commercially available RT-PCR kits (Xiamen Zeesan Biotech Co., Ltd., Fujian, China) were used for the detection of MTB. The assay specifically targets the *IS6110* insertion element, which is unique to *Mycobacterium tuberculosis*. The RT-PCR assay was performed on the automated medical PCR analysis system Sanity 2.0 (Xiamen Zeesan Biotech Co., Ltd., Fujian, China). Briefly, each clinical sample was mixed with an equal volume of sample treatment solution and vortexed for 30 seconds. After complete liquefaction, 1 mL of the mixture and 10 μL of enhancement solution were transferred into the sample chamber of a disposable nucleic acid extraction and purification cartridge. The cartridge and a pre-loaded PCR reaction tube were then loaded into the analyzer. The entire process—including DNA extraction, purification, and real-time PCR amplification—was performed automatically by the instrument. In accordance with our laboratory’s quality control protocol, positive and negative controls provided with the kit were run once daily. The thermal cycling conditions pre-programmed in the analyzer were as follows: initial denaturation at 95 °C for 2 min; followed by 10 cycles of touchdown PCR (95 °C for 7 s, 65 °C for 15 s, with a decrease of 1 °C per cycle); and then 40 cycles of amplification (95 °C for 7 s, 55 °C for 15 s). Fluorescence signals were collected in the FAM and HEX channels at the end of each annealing/extension step. In the case of a positive test, a cycle threshold (Ct) value was provided. The Ct value is the PCR cycle number at which the fluorescence signal crosses the detection threshold. A lower Ct value indicates a higher amount of the target nucleic acid in the sample. According to the manufacturer’s instructions, samples with a FAM channel Ct value ≤25 were interpreted as positive for MTB. Samples with a Ct value >25 or no amplification signal, coupled with a HEX channel (internal control) Ct value <26, were scored as negative. An invalid result was indicated if the internal control failed.

### The Xpert MTB/RIF assay

Xpert MTB/RIF assay (Cepheid, Sunnyvale, CA, USA) is a fully integrated, heminested real-time PCR assay performed on the GeneXpert system (Cepheid, Sunnyvale, CA, USA). The assay simultaneously detects the presence of MTB DNA and mutations associated with rifampin resistance within an 81 bp region of the *rpoB* gene (the rifampin resistance-determining region, RRDR). According to the manufacturer’s protocol, a 2 : 1 volume of sample reagent (SR) buffer was added to the cryopreserved sample. The mixture was vortexed for 20 seconds and incubated at room temperature for 15 minutes. Subsequently, 2 mL of the processed sample was transferred into the Xpert MTB/RIF assay cartridge using a disposable pipette. The cartridge was then loaded into the GeneXpert instrument. The GeneXpert system automatically performed sample lysis, DNA extraction, purification, and PCR amplification. The specific primers and five molecular beacon probes (CF1, CF3, CF4, CF5, CF6) targeting the *rpoB* gene are proprietary and pre-loaded in the cartridge. The PCR incubation and fluorescent detection are integral parts of the closed system and were carried out according to the pre-programmed protocol. Each cartridge contains internal controls: a sample processing control (SPC) to monitor extraction and inhibition, and a probe check control (PCC) to verify reagent rehydration, probe integrity, and dye stability. The instrument software automatically analyzed the fluorescence data and reported results as MTB “NOT DETECTED” or “DETECTED” with semi-quantification (Extremely Low, Low, Medium, High). Rifampin resistance was reported as “DETECTED”, “NOT DETECTED”, or “INDETERMINATE”.

### Validation of mNGS Results

In an effort to orthogonally validate the mNGS findings, we attempted Sanger sequencing on the residual frozen samples for all discordant cases. However, due to the limited volume of cryopreserved specimens remaining after the initial mNGS, RT-PCR, and Xpert MTB/RIF assays, only one sample (Case 7, a bronchoalveolar lavage fluid) had sufficient material for this additional analysis. DNA from this sample was amplified using primers (TR8x, 5′-TCGCCGCGATCAAGGAGTTCTTCGGC-3′; TR9x, 5′-TGCACGTCGCGGACCTCCAGCCCGGCAC-3′) targeting the *rpoB* gene (Yam et al., 2004), followed by Sanger sequencing. VAMNE Magnetic Pathogen DNA/RNA Kit (Vazyme Biotech Co., Ltd., China) was used for DNA extraction according to the manufacturer’s instruction. DNA amplification was performed in a thermocycler nexus gradient (Eppendorf, Germany), in a final volume of 50 µL containing 2 x Phanta UniFi Master Mix (Vazyme Biotech Co., Ltd., China), 0.4 µM of each primer, and 5 µL of template DNA. The amplification program was consisted of initial denaturation at 98 °C for 30 s, followed by 35 cycles of denaturation at 98 °C for 10 s, annealing at 65 °C for 10 s, extension at 72 °C for 15 s, and a final extension at 72 °C for 5 min. After PCR amplification, a 2 µL aliquot of the PCR product was electrophoresed for 30 min through 1.5% agarose gel, and the target band of 157-bp was visualized under UV illumination. The 157-bp fragment was subjected to sequencing on 3730 Genetic Analyzer (Applied Biosystems, USA). The resulting sequence was analyzed using the BLASTn algorithm against the NCBI nucleotide database.

### Statistical analysis

Continuous variables were presented as medians and interquartile ranges, while categorical variables were expressed as frequencies and percentages. The Cohen’s *kappa* test was used to assess agreement between the two testing methods, and Spearman’s correlation analysis was performed to examine the relationship between SMRNs and Ct values. The Mann-Whitney *U* test was conducted to compare the Ct values and SMRNs between the concordant and discordant groups. All statistical tests were two-sided, and *P* value <0.05 were considered as statistically significant. IBM SPSS 17.0 software was used for statistical analysis.

## Results

### Sample characteristics

This study enrolled 556 patients. The median age was 61 years (interquartile range [IQR]: 49; 68), and 65.11% of the participants were male, while 34.89% were female. The most common comorbidities in our cohort were hypertension (50.18%) and type 2 diabetes mellitus (39.21%). The majority of samples (97.30%) were obtained from the lower respiratory tract, including 523 bronchoalveolar lavage fluid (BALF) samples (94.06%) and 18 sputum samples (3.24%). Extrapulmonary samples accounted for 2.70% of the total, consisting of 11 puncture fluids (1.98%), 2 tissue biopsies (0.36%), 1 urine sample (0.18%), and 1 lymph node sample (0.18%) (**Table 1**).

**Table 1:** Baseline characteristics and sample types of the study cohort.

Characteristics	All patients (n=556)
Demographics	
Age, years, median (IQR)	61 (49; 68)
Male, n (%)	362 (65.11)
Underlying diseases/comorbidities, n (%)	
Cardiovascular diseases	
Hypertension	279 (50.18)
Coronary heart disease	70 (12.59)
Heart failure	47 (8.45)
Myocardial infarction	7 (1.26)
Arrhythmia	21 (3.78)
Atrial fibrillation	35 (6.29)
Metabolic diseases	
Type 2 diabetes mellitus	218 (39.21)
Hyperlipidemia	85 (15.29)
Hyperuricemia	73 (13.13)
Respiratory diseases	
Pneumonia	129 (23.20)
Chronic obstructive pulmonary disease (COPD)	49 (8.81)
Bronchiectasis	48 (8.63)
Lung cancer	32 (5.76)
Pulmonary nodule	76 (13.67)
Renal diseases	
Chronic kidney disease	109 (19.60)
Nephrotic syndrome	11 (1.98)
Lupus nephritis	8 (1.44)
Malignancies (Non-pulmonary)	
Leukemia	27 (4.86)
Lymphoma	30 (5.40)
Multiple myeloma	26 (4.68)
Gastric cancer	6 (1.08)
Colorectal cancer	9 (1.62)
Breast cancer	9 (1.62)
Prostate cancer	10 (1.80)
Digestive system diseases	
Fatty liver disease	75 (13.49)
Liver cirrhosis	34 (6.12)
Gallstones	33 (5.94)
Gallbladder polyps	3 (0.54)
Gastritis	25 (4.50)
Esophagitis	8 (1.44)
Hematologic diseases	
Anemia	78 (14.03)
Thrombocytopenia	44 (7.91)
Myelodysplastic syndromes (MDS)	13 (2.34)
Autoimmune diseases	
Systemic lupus erythematosus (SLE)	20 (3.60)
ANCA-associated vasculitis	14 (2.52)
Rheumatoid arthritis	6 (1.08)
Hepatic diseases	
Liver cirrhosis	34 (6.12)
Hepatic insufficiency	24 (4.32)
Hepatic cyst	70 (12.59)
Neurological diseases	
Cerebral infarction	39 (7.01)
Brain atrophy	52 (9.35)
Parkinson’s disease	6 (1.08)
Symptomatic epilepsy	3 (0.54)
Infectious diseases	
Tuberculosis	13 (2.34)
Hepatitis B	41 (7.37)
Invasive fungal disease	7 (1.26)
Endocrine diseases	
Thyroid dysfunction	31 (5.58)
Adrenal gland diseases	5 (0.90)
Pituitary gland diseases	5 (0.90)
Skeletal system diseases	
Osteoporosis	29 (5.22)
Fracture	16 (2.88)
Intervertebral disc disease	11 (1.98)
Bone metastasis	10 (1.80)
Others	
Benign prostatic hyperplasia	33 (5.94)
Cataract	7 (1.26)
Glaucoma	3 (0.54)
Sample type, n (%)	
Lower respiratory tract samples	541 (97.30)
Bronchoalveolar lavage fluid (BALF)	523 (94.06)
Sputum	18 (3.24)
Extrapulmonary samples	15 (2.70)
Puncture fluid	11 (1.98)
Tissue	2 (0.36)
Urine	1 (0.18)
Lymph node	1 (0.18)

n: number of cases.

IQR, interquartile range.

### Concordance between mNGS and RT-PCR for MTB detection

To assess the concordance between mNGS and RT-PCR for detection of MTB, we analyzed the results of 556 samples using Cohen’s *kappa* coefficient. The results indicated that among RT-PCR-negative samples, mNGS was negative in 504 cases and positive in 5 cases; among RT-PCR-positive samples, mNGS was negative in 4 cases and positive in 43 cases. The agreement analysis yielded a positive agreement (PA) of 82.69%, negative agreement (NA) of 98.25%, and an overall agreement (ORA) of 98.38%. The *kappa (κ)* value was 0.896 (95% CI: 0.830-0.963, *P* < 0.001), demonstrating a high level of concordance between the two methods for MTB detection (**Table 2**).

**Table 2:** Consistency between mNGS and RT-PCR.

RT-PCR	mNGS		PA, %	NA, %	ORA, %	*κ*	*P*
	+	-	(95% CI)	(95% CI)	(95% CI)	(95% CI)	
+	43	4	82.69	98.25	98.38	0.896	<0.001
–	5	504	(69.67-91.77)	(96.70-99.20)	(96.95-99.26)	(0.830-0.963)	

+, positive; -, negative; PA, positive agreement; NA, negative agreement; ORA, overall agreement.

### Concordance between mNGS and RT-PCR based on Ct value stratification

For samples with Ct values ≤20, complete agreement (100%) was observed between the two detection methods. Method discrepancies occurred mainly in samples with Ct values between 20 and 30 (**Table 3**).

**Table 3:** Concordance between mNGS and RT-PCR, stratified by RT-PCR Ct values.

Ct values	PA, %	NA, %	95% CI
≤15	100(9/9)	N/A	66.37-100
15<Ct ≤ 20	100(21/21)	N/A	83.89-100
20<Ct ≤ 25	76.47(13/17)	N/A	50.10-93.19
25<Ct ≤ 30	N/A	90.91(20/22)	70.84-98.88
>30	N/A	100(6/6)	54.07-100
NoCt	N/A	99.38(478/481)	98.19-99.87

Ct, cycle threshold; PA, positive agreement; NA, negative agreement.

PA and NA could not be calculated (N/A) for strata with no RT-PCR-positive or no RT-PCR-negative samples, respectively.

### Correlation analysis between mNGS SMRNs and RT-PCR Ct values

**Figure 1:**
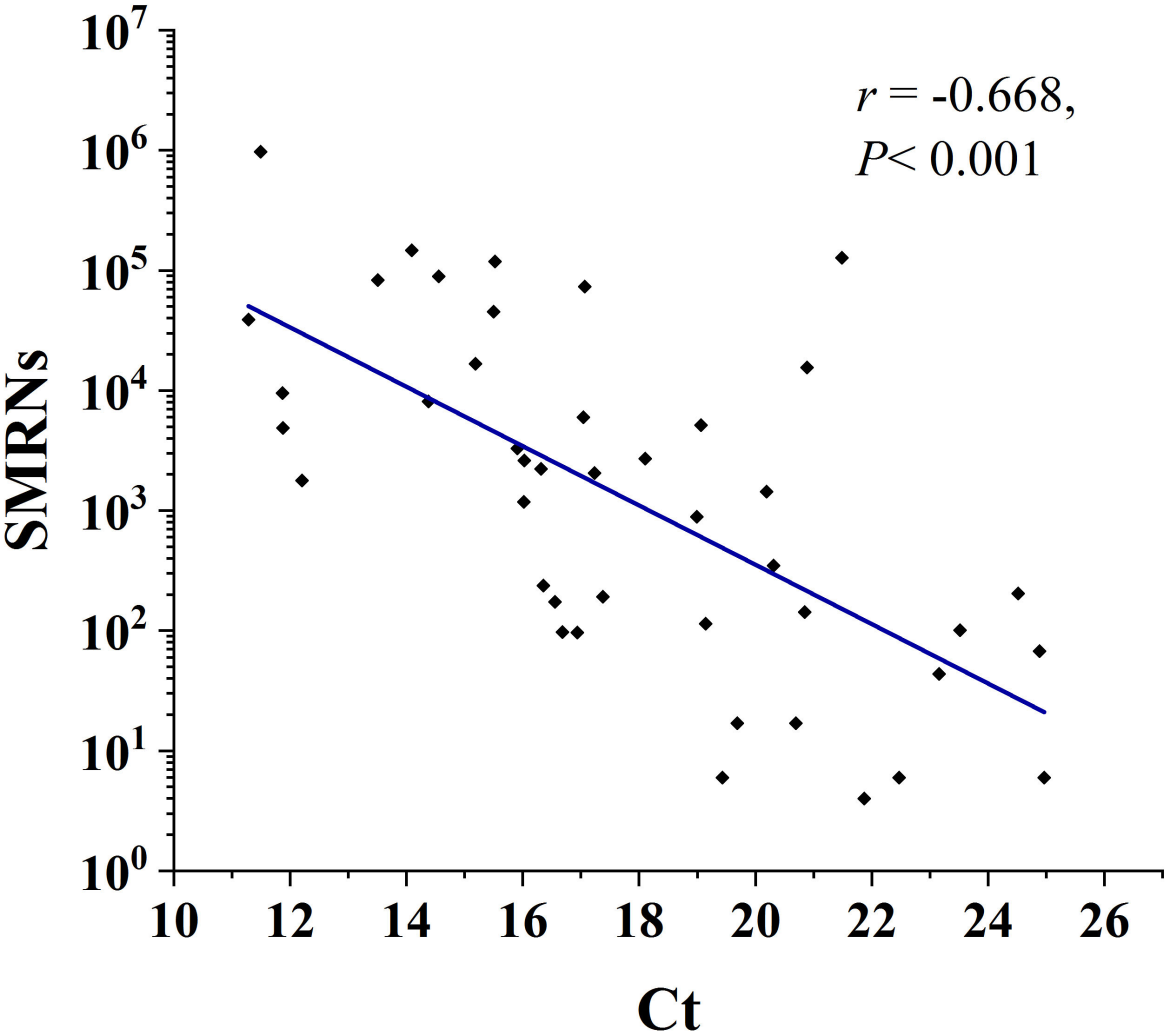
Correlation between standardized microbial read numbers (SMRNs) from mNGS and cycle threshold (Ct) values from RT-PCR. Scatter plot showing a strong negative correlation (*r* = -0.668, *P* < 0.001) between SMRNs (the number of sequencing reads uniquely mapped to *Mycobacterium tuberculosis*) and Ct values among the 43 samples that were positive by both methods.

Among the 43 samples that tested positive by both methods, scatter plot analysis revealed a clear decreasing trend in SMRNs— from 10_7_; to 10_0_ — as Ct values increased from 10 to 25, suggesting a strong negative correlation (*r* = -0.668, *P* < 0.001) between SMRNs and Ct values (**Figure 1**).

### Analysis of inconsistent cases

**Figure 2:**
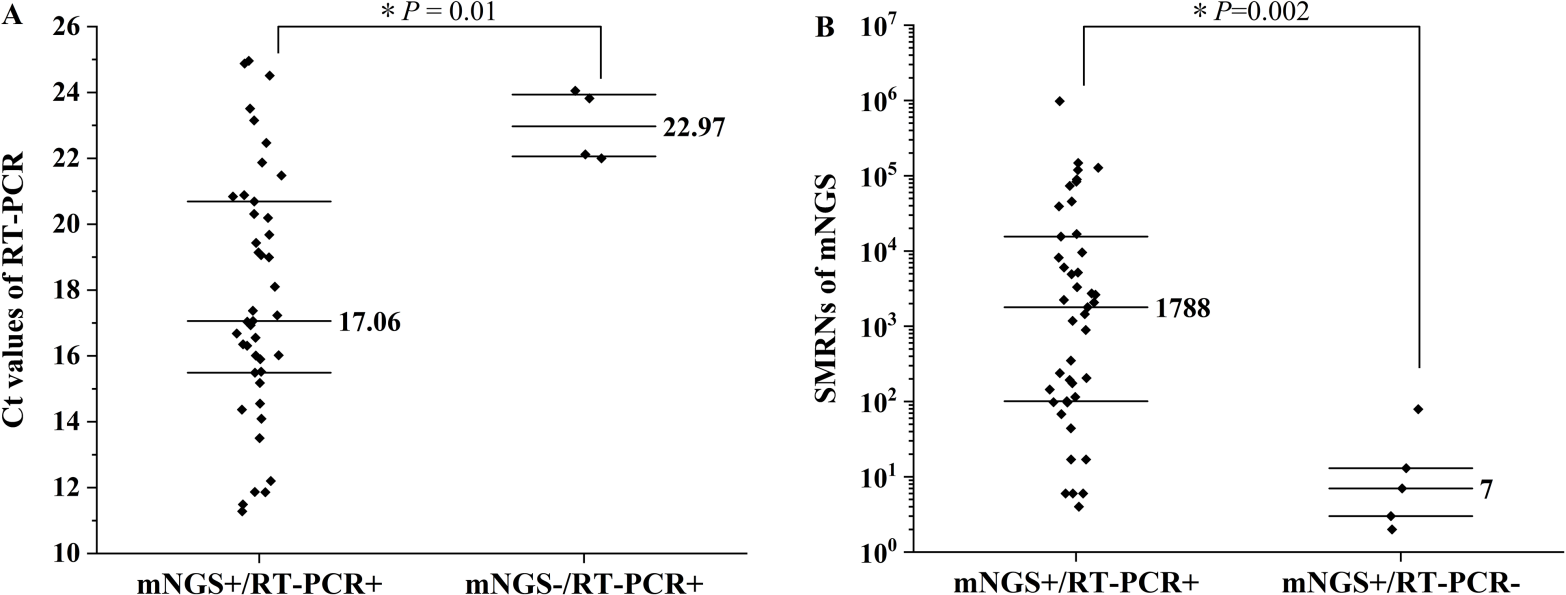
Distribution of cycle threshold (Ct) values and standardized microbial read numbers (SMRNs) in concordant and discordant samples. **(A)** Comparison of RT-PCR Ct values between concordant positive (mNGS+/RT-PCR+, n=43) and mNGS-negative/RT-PCR-positive (mNGS/RT-PCR+, n=4) samples (*Z* = -2.59, *P* = 0.01). **(B)** Comparison of mNGS SMRNs between concordant positive (mNGS+/RT-PCR+, n=43) and mNGS-positive/RT-PCR-negative (mNGS+/RT-PCR-, n=5) samples (*Z* = -3.09, *P* = 0.002). * indicates a statistically significant difference between the groups (*P* < 0.05).

The Xpert MTB/RIF assay was applied to nine samples with discordant results between mNGS and RT-PCR. Among the four mNGS-negative/RT-PCR-positive cases, the median Ct value (22.97) was higher than that of the concordant positive cases (17.06) [*Z* = -2.59, *P* = 0.01] (**Figure 2A**). Xpert testing returned “extremely low concentration” for three of these samples, while one was negative. Of the five mNGS-positive/RT-PCR-negative cases, the median SMRNs (7) was significantly lower than that in double-positive samples (1788) [*Z* = -3.09, *P* = 0.002] (**Figure 2B**). Xpert results indicated “extremely low concentration” in four cases; the remaining sample, a lymph node aspirate, tested negative. Eight of these cases were clinically confirmed as tuberculosis, although one outpatient case could not be definitively diagnosed owing to insufficient clinical data (**Table 4**). Furthermore, to orthogonally validate the mNGS result, we performed Sanger sequencing of the *rpoB* gene on the available residual sample (Case 7). A BLASTn search of the obtained sequence against the NCBI nucleotide database revealed that the top hit was *Mycobacterium tuberculosis* (GenBank accession no. HM776957.1), with 100% sequence identity and 95% query coverage, thereby confirming the specific mNGS finding in this case. Sequence information was provided in **Supplementary Material**.

**Table 4:** Summary of 9 cases with discordant mNGS and RT-PCR results.

No.	Sample	RT-PCR (Ct)	mNGS (SMRNs)	Xpert (concentration)	Clinical diagnosis
1	BALF	+ (22.00)	–	Extremely low	Pulmonary TB, after 6 months of ATT
2	BALF	+ (23.82)	–	Extremely low	Pulmonary TB, after 3 months of ATT
3	BALF	+ (22.12)	–	Extremely low	Previous history of TB
4	BALF	+ (24.05)	–	–	Pulmonary TB
5	BALF	- (NoCt)	+ (79)	Extremely low	Pulmonary TB
6	BALF	- (NoCt)	+ (3)	Extremely low	Pulmonary TB
7	BALF	- (27.05)	+ (7)	Extremely low	Pulmonary TB
8	BALF	- (28.60)	+ (13)	Extremely low	Not applicable
9	Lymph node	- (NoCt)	+ (2)	–	TP, TBP, after 2 weeks of ATT

Ct, cycle threshold; SMRNs, strict mapped read numbers; BALF, bronchoalveolar lavage fluid; +, positive; -, negative; TB, tuberculosis; ATT, anti-tuberculous therapy; TP, tuberculous pleuritis; TBP, tuberculous pericarditis.

Sanger sequencing of the *rpoB* gene fragment was successfully performed on the residual sample from Case 7. BLAST analysis of the obtained sequence confirmed the presence of Mycobacterium tuberculosis DNA.

### Diagnostic performance of mNGS and RT-PCR for TB

To assess the diagnostic performance of mNGS and RT-PCR for tuberculosis (TB), both methods were evaluated against a composite reference standard consisting of Xpert MTB/RIF results and final clinical diagnosis. The analysis revealed that mNGS had a sensitivity of 92.31% (48/52) and a specificity of 100% (504/504), whereas RT-PCR exhibited a sensitivity of 90.38% (47/52) and a specificity of 100% (504/504) (**Table 5**).

**Table 5:** Diagnostic performance and agreement with the composite reference standard for mNGS and RT-PCR.

Method		Attributed by Xpert and clinical diagnosis		Sensitivity	Specificity	ORA
		TB	Non-TB	(95% CI)	(95% CI)	(95% CI)
mNGS	+	48	0	92.31	100	99.28
	–	4	504	(81.46-97.86)	(99.27-100)	(98.17-99.80)
RT-PCR	+	47	0	90.39	100	99.10
	–	5	504	(78.97-96.80)	(99.27-100)	(97.91-99.71)

TB, tuberculosis; ORA, overall agreement; +, positive; -, negative.

Note: A positive result on the Xpert assay or a clinical diagnosis of tuberculosis is considered the standard for diagnosing tuberculosis.

It should be noted that the number of confirmed TB-positive cases in our cohort was relatively small (n=52), which is a limitation for the precise estimation of diagnostic accuracy metrics. While the high sensitivity and specificity observed are encouraging, these point estimates should be interpreted with caution, and their confidence intervals should be considered. The findings require validation in larger, prospective studies.

## Discussion

MTB primarily infects the lungs but can also disseminate to extrapulmonary sites such as lymph nodes, bones, the genitourinary tract, and the pleura, representing a major challenge to global public health. Although etiological examination remains the cornerstone of TB diagnosis, only 62% of the estimated 6.9 million global active TB cases in 2023 were bacteriologically confirmed (Global tuberculosis report, 2024). This underscores the urgent need to transition from conventional techniques to molecular diagnostics, which offer substantially improved sensitivity (Pai et al., 2023). In this study, we compared the diagnostic performance and agreement between mNGS and RT-PCR for detecting MTB, aiming to provide evidence-based guidance for clinical practice.

In our evaluation of 556 clinically suspected TB cases, mNGS demonstrated a slightly higher sensitivity (92.31%) than RT-PCR (90.38%), while both methods achieved 100% specificity. These results reflect strong diagnostic efficacy and are consistent with recent studies (Wei et al., 2019; Li et al., 2023), supporting the value of molecular approaches in TB diagnosis.

Excellent overall agreement was observed between mNGS and RT-PCR (ORA = 98.38%, *κ* = 0.896). A significant inverse correlation was identified between standardized microbial read numbers (SMRNs) and cycle threshold (Ct) values among concordant positive samples. Theoretically, SMRNs correlate positively with the nucleic acid burden of target pathogens in clinical specimens. Higher microbial burdens in samples increase pathogen-derived nucleic acids in extracts, consequently yielding substantially more SMRNs during sequencing. Conversely, samples with higher initial target nucleic acid concentrations require fewer cycles to reach the fluorescence threshold during PCR amplification, resulting in lower Ct values. Thus, both SMRNs and Ct values serve as reliable biomarkers for estimating relative MTB burden. Consequently, samples with high MTB load demonstrate elevated SMRNs by mNGS alongside reduced Ct values by RT-PCR, establishing a robust cross-validation relationship between the two techniques.

However, the positive agreement rate (82.69%) between the two methods was suboptimal. Stratification by Ct value revealed complete concordance (100%) in specimens with Ct ≤ 20, whereas discrepancies predominantly occurred in high-Ct samples (Ct>20). Among 17 samples with 20<Ct ≤ 25, four BLAF specimens detected negative for MTB by mNGS: three were Xpert-positive, and one was Xpert-negative but clinically diagnosed as TB. This four mNGS-negative/RT-PCR-positive cases had significantly higher median Ct values than the double-positive group (22.97 vs 17.06, *P* < 0.05), suggesting that low bacterial burden contributed to the discrepancies. Respiratory specimens commonly contain substantial host-derived nucleic acids, potentially interfering with the detection of low-prevalence pathogens (Diao et al., 2022). Host DNA depletion strategies have been shown to improve mNGS sensitivity in BLAF samples (Yuan et al., 2025). Additionally, two discordant cases had received over 3 months of anti-TB treatment. Prior research suggests that prolonged treatment (>3 months) reduces bacterial load and mNGS sensitivity (Liu et al., 2021), which may explain these observations.

Among the five mNGS-positive/RT-PCR-negative cases, four exhibited very low SMRNs and were confirmed by Xpert at extremely low concentrations, indicating probable false-negative RT-PCR results due to low MTB burden. The RT-PCR assay used in this study targets the *IS6110* element, whose copy number varies widely across strains (0~25 copies) (Couvin et al., 2025). False negatives may occur in strains with low or absent *IS6110* copies. In contrast, mNGS targets conserved genomic regions, circumventing issues related to sequence variability. Notably, one lymph node aspirate with only 2 SMRNs was negative by Xpert but clinically confirmed as tuberculous pleuritis and pericarditis. Given that MTB is an intracellular pathogen and DNA extraction is challenging, even a single SMRN may indicate MTB infection (Zhang et al., 2023). Previous studies report superior sensitivity of mNGS over Xpert for lymph node TB (89.47% vs. 68.42%) (Lin et al., 2025), supporting its utility in extrapulmonary and paucibacillary samples (Yan et al., 2020; Huang et al., 2023).

mNGS and RT-PCR show high concordance and are mutually validating. For samples near the detection limit, combined use of both methods is recommended to avoid missed diagnoses. RT-PCR offers rapid turnaround (∼3 hours) and lower cost, making it suitable for acute settings. Conversely, mNGS requires approximately 24 hours but provides unparalleled advantage in detecting extrapulmonary TB and co-infections. TB patients are susceptible to polymicrobial infections due to immune suppression and tissue damage (Bir et al., 2024; Muni et al., 2023), and mNGS enables comprehensive pathogen screening in a single run (Liu et al., 2021; Yuan et al., 2024). To balance cost and efficacy, we propose RT-PCR as first-line testing for suspected TB, with mNGS reserved for RT-PCR-negative cases with borderline Ct values, persistent symptoms, or suspected extrapulmonary/complex infections.

This study has several limitations. First, its retrospective design and the use of cryopreserved samples for the additional Xpert MTB/RIF verification represent a constraint. Although storage at −80 °C is a standard practice, we cannot entirely rule out the potential for analyte degradation. However, it is crucial to note that the diagnostic performance of mNGS and RT-PCR was evaluated against a composite reference standard that incorporated both Xpert results and the final clinical diagnosis, which was based on a comprehensive assessment of fresh clinical data, treatment response, and radiological findings. This clinical diagnosis is the ultimate arbiter in practice and is less susceptible to the effects of sample storage. Therefore, while a prospective fresh-sample study is the ideal for validating absolute sensitivity, our use of a robust clinical composite standard ensures that our comparative findings regarding the agreement of mNGS and RT-PCR remain valid and clinically relevant. Another significant limitation of this study is the relatively small number of TB-positive cases (n=52), which limits the statistical power and precision of our sensitivity estimates. A sample size with a low event rate can lead to overfitting and potentially overoptimistic performance metrics. The wide confidence intervals for sensitivity (e.g., 81.46%-97.86% for mNGS) underscore this uncertainty. Therefore, our primary conclusions should be focused on the high concordance between mNGS and RT-PCR, which was the main objective, and the complementary role they may play in diagnosis, particularly in challenging cases with low bacterial loads. The promising diagnostic performance observed here must be rigorously validated in larger, multi-center cohorts with a higher prevalence of confirmed TB. Additionally, the predominance of pulmonary samples (97.48%) limits generalizability to extrapulmonary TB. Future studies should include more diverse sample types to validate method concordance.

The observed spectrum of bacterial loads and the number of confirmed TB cases in our cohort must be interpreted within the specific clinical context of TB diagnosis in China. As a country with a high TB burden, China’s public health strategy mandates active screening of suspected cases. In this framework, patients with classic symptoms and signs are often successfully diagnosed using conventional, cost-effective methods such as smear microscopy and culture. Our study cohort, however, is comprised of patients for whom physicians deemed it necessary to simultaneously employ two advanced molecular tests (mNGS and RT-PCR). This typically occurs in scenarios involving diagnostically challenging cases, such as those with atypical clinical presentations, suspected paucibacillary disease, immunosuppression, or complex comorbidities. Therefore, the relatively lower TB positivity rate in our study, compared to broader epidemiological surveys, is not a weakness but rather an accurate reflection of the real-world application of these technologies. It precisely captures their utility in the most difficult-to-diagnose patient subgroups. Consequently, the high agreement and diagnostic performance we observed between mNGS and RT-PCR are highly relevant and validate their complementary role in resolving complex clinical dilemmas.

In conclusion, this study validates a high concordance between mNGS and RT-PCR for detecting MTB in a real-world patient cohort. Their complementary strengths—particularly in cases with low bacterial loads—provide a robust molecular strategy for guiding the diagnosis of clinically complex tuberculosis.

## Data Availability

The original contributions presented in the study are included in the article/**Supplementary Material**. Further inquiries can be directed to the corresponding author.
